# Magnetic Resonance Imaging and Histopathological Visualization of Human Dural Lymphatic Vessels

**DOI:** 10.21769/BioProtoc.2819

**Published:** 2018-04-20

**Authors:** Seung-Kwon Ha, Govind Nair, Martina Absinta, Nicholas J. Luciano, Daniel S. Reich

**Affiliations:** Division of Neuroimmunology and Neurovirology, National Institute of Neurological Disorders and Stroke (NINDS), National Institutes of Health (NIH), Bethesda, MD, USA

**Keywords:** Lymphatic vessels, Brain, Meninges, MRI, Histopathology, Immunohistochemistry

## Abstract

In this protocol, we describe a method to visualize and map dural lymphatic vessels *in-vivo* using magnetic resonance imaging (MRI) and *ex-vivo* using histopathological techniques. While MRI protocols for routine imaging of meningeal lymphatics include contrast-enhanced T2-FLAIR and T1- weighted black-blood imaging, a more specific 3D mapping of the lymphatic system can be obtained by administering two distinct gadolinium-based MRI contrast agents on different days (gadofosveset and gadobutrol) and subsequently processing images acquired before and after administration of each type of contrast. In addition, we introduce methods for optimal immunostaining of lymphatic and blood vessel markers in human dura mater *ex-vivo*.

## Background

Among the causes of immune privilege in the brain is the absence of parenchymal lymphatic vessels. However, recent studies have uncovered an extensive lymphatic circulating system in the dura mater of rodents (Aspelund *et al*., 2015; Louveau *et al*., 2015), providing possible routes for the elimination of the brain’s waste products and for immune cells to access the deep cervical lymph nodes. In this protocol, we describe a way to: (1) visualize the lymphatic vessels *in-vivo* in the dura mater using MRI of the head, and (2) assess the local presence of lymphatic vessels using optimized immunostaining methods (Absinta *et al.*, 2017). *In-vivo* imaging of lymphatics may enable more detailed studies of mechanisms of waste removal and immune function and their potential abnormalities in various diseases and aging.

## Materials and Reagents

1. Superfrost Plus Microslides (Daigger Scientific, catalog number: EF15978Z)
2. Cover Glasses (Daigger Scientific, catalog number: EF15972L)
3. Paper towel (KCWW, Kimberly-Clack, catalog number: 05511)
4. Polypropylene Coplin jar (IHC World, catalog number: IW-2501)
5. Super HT PAP pen (Biotium, catalog number: 22006)
6. Gadavist, gadobutrol (0.1 mmol/kg body weight, i.v., Bayer Health Care, NDC 50419-325-12)
7. Ablavar, gadofosveset (0.03 mmol/kg body weight, i.v., Lantheus Medical Imaging, NDC 11994- 012-02)
8. 10% Neutral Buffered Formalin Fixatives, methanol < 2% (Leica Biosystems, catalog number: 3800602)
9. Ethanol (Pharmaco-AAPER, catalog number: 111000200)
10. Target Retrieval Solution, pH 9 (Agilent Technologies, Dako, catalog number: S2367)
11. Target Retrieval Solution (Agilent Technologies, Dako, catalog number: S1699)
12. Tris buffered saline 10×, pH 7.4 (KD Medical, catalog number: RGF-3385)
13. Hydrogen Peroxide, 30% (Fisher Scientific, catalog number: H325-500)
14. Protein Block, Serum-Free (Agilent Technologies, Dako, catalog number: X0909)
15. LYVE1 antibody (Abcam, catalog number: ab36993)
16. Podoplanin (D2-40) antibody (Bio-Rad Laboratories, catalog number: MCA2543)
17. CD31 antibody (Abcam, catalog number: ab28364)
18. PROX1 antibody (AngioBio, catalog number: 11-002P)
19. COUP-TF II antibody (R&D Systems, catalog number: PP-H7147-00)
20. CCL21 antibody (Abcam, catalog number: ab9851)
21. CD68 (KP-1) antibody (Thermo Fisher Scientific, Invitrogen, catalog number: MA5-13324)
22. CD3 antibody (Agilent Technologies, Dako, catalog number: A0452)
23. Antibody Diluent (Agilent Technologies, Dako, catalog number: S0809)
24. PV Poly-HRP Anti-Mouse IgG (Leica Biosystems, catalog number: PV6114)
25. PV Poly-HRP Anti-Rabbit IgG (Leica Biosystems, catalog number: PV6119)
26. ImmPRESS™-AP Anti-Rabbit IgG (Vector Laboratories, catalog number: MP-5401)
27. ImmPRESS™-AP Anti-Mouse IgG (Vector Laboratories, catalog number: MP-5402)
28. Goat anti-Mouse IgG, Alexa Fluor 488 (Thermo Fisher Scientific, Invitrogen, catalog number: A- 11029)
29. Goat anti-Rabbit IgG, Alexa Fluor 594 (Thermo Fisher Scientific, Invitrogen, catalog number: A- 11012)
30. DAB substrate kit (Abcam, catalog number: ab94665)
31. Vector Blue Alkaline Phosphatase Substrate Kit (Vector Laboratories, catalog number: SK-5300)
32. Hematoxylin 560 MX (Leica Biosystems, catalog number: 3801575)
33. Blue buffer 8 (Leica Biosystems, catalog number: 3802916)
34. VectaMount Permanent Mounting Medium (Vector Laboratories, catalog number: H-5000)
35. Fluoro-Gel II Mounting Medium (Electron Microscopy Sciences, catalog number: 17985-50)
36. Tween 20 (Agilent Technologies, Dako, catalog number: S1966)
37. TBS-0.5% Tween 20 (TBST) (see Recipes)

## Equipment

1. 18-22 gauge catheter (Smiths Medical)
2. Pressure infusion tubing (ICU Medical)
3. Automatic pressure injector (Bayer, model: Medrad^®^ Spectris Solaris^®^ EP MR Injection System)
4. 3-tesla MRI scanner unit (Siemens Skyra, Siemens Healthcare)
5. 32-channel head coil for MRI signal reception (Siemens Skyra, Siemens Healthcare)
6. Water bath (Leica Biosystem, model: Leica HI1210)
7. Humidified chamber (Simport, model: StainTray™ M920)
8. Manual Rotary Microtome (Leica Biosystem, model: Leica RM2235)
9. Leica RM CoolClamp™ (Leica Biosystem, model: Leica RM CoolClamp)
10. Steamer (IHC World, model: IHC-Tek™ Epitope Retrieval Steamer Set)
11. Digital rocker (VWR, catalog number: 12620-906)
12. Microscope (Carl Zeiss, model: AxioObserver Z.1)
13. Microscope camera (Carl Zeiss, model: Axiocam 503)
14. Magnetic stirrer

## Software

1. MIPAV software (https://mipav.cit.nih.gov/)
2. OsiriX software (http://www.osirix-viewer.com/)
3. Zeiss Zen 2 Blue edition (https://www.zeiss.com/microscopy/int/products/microscope- software/zen.html)

## Procedure

### A. Ethical approval

All human research was carried out under an Institutional Review Board approved protocol, after obtaining informed consent. Formalin-fixed human dura was retrieved at autopsy after obtaining appropriate consent.

### B. Human imaging

**Figure 1:**
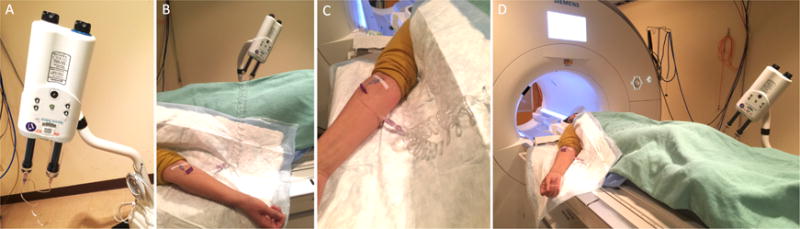
MRI preparation Auto injector setup showing the injector (A), linked to the catheter through extension tubing (B, C), and the setup before the subject is moved into the MRI for scanning (D).

**Figure 2:**
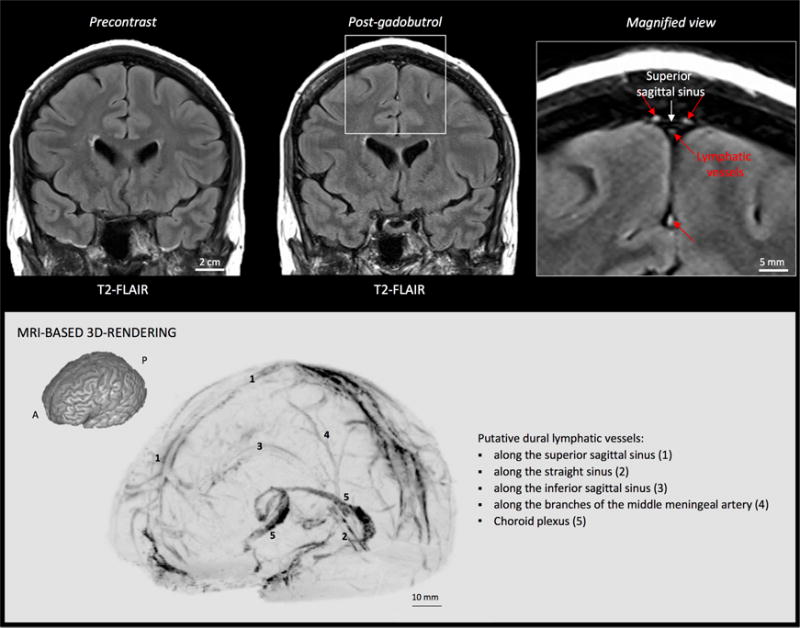
MRI visualization of dural lymphatic vessels in human On post-gadobutrol coronal T2-FLAIR, the dura does not enhance, and lymphatic vessels (red arrows), running alongside the venous dural sinuses and within the falx cerebri, can be appreciated. 3D rendering, using OsiriX software, of putative dural lymphatics (black) in a 47-year old woman, derived from whole-brain T1-weighted SPACE MRI. (Modified from Figure 1 and Figure S1 in Absinta *et al*. [2017]. Creative Commons Attribution License)

**Figure 3:**
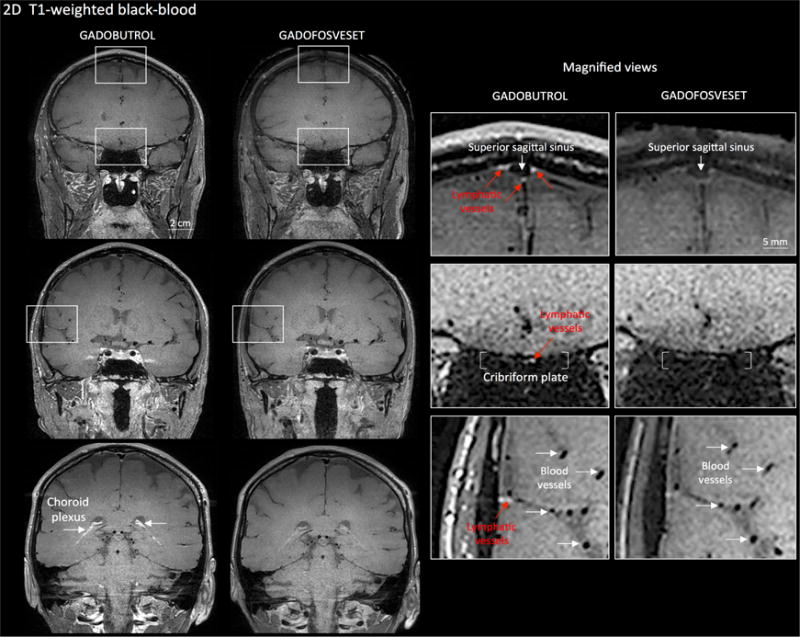
Gadobutrol vs. gadofosveset in MRI visualization of dural lymphatic vessels Coronal T1-weighted black-blood images were acquired after intravenous injection of two different gadolinium-based contrast agents during two MRI sessions separated by one week. Dural lymphatics (red arrows in magnified view boxes) were better discerned using gadobutrol (standard MRI contrast agent, which readily enters the dura) compared to gadofosveset (serum albumin-binding contrast agent, which remains largely intravascular) and were localized around dural sinuses, middle meningeal artery, and cribriform plate (white arrows). Notably, the choroid plexus (white arrows) enhanced less with gadofosveset than gadobutrol, whereas meningeal and parenchymal blood vessels (both veins and arteries) did not enhance with any contrast agent and appeared black. (Originally published in Absinta *et al*. [2017]. Creative Commons Attribution License)

1. Place an intravenous line using an 18-22 gauge catheter and pressure infusion tubing linked to an MRI compatible automatic pressure injector. (Figure 1)
2. Set up the subject in the MRI scanner with a 32-channel head coil.
3. Perform cranial MRI as following sequences, a quoted method from Absinta *et al.* (2017).
  - Whole-brain T1-Magnetization Prepared Rapid Acquisition of Gradient Echoes (MPRAGE, sagittal 3D turbo-fast low angle shot sequence, acquisition matrix 256 × 256, isotropic resolution 1 mm, 176 slices, repetition time [TR]/echo time [TE]/inversion time [TI] = 3,000/3/900 msec, flip angle 9°, acquisition time 5 min 38 sec).
  - Limited T2-weighted Fluid Attenuation Inversion Recovery (FLAIR, coronal 2D acquisition over the superior sagittal sinus, field-of-view 256 mm^2^, 22 slices, reconstructed in-plane resolution 0.25 mm^2^, 42 contiguous 3 mm slices, TR/TE/TI = 6,500/93/2,100 msec, echo train length 17, bandwidth 80 Hz/pixel, acquisition time 5 min), optimized for detection of gadolinium-based contrast agent in the subarachnoid space.
  - Black-blood scan (coronal acquisition, Sampling Perfection with Application optimized Contrasts using different flip angle Evolution [SPACE] sequence, field-of-view 174 mm^2^, matrix 320 × 320, reconstructed in-plane resolution 0.27 mm^2^, 64 contiguous 0.5 mm sections, TR/TE = 938/22 msec, echo train length 35, bandwidth 434 Hz/pixel, acquisition time 7 min 50 sec). Acquire a series of 2 or three overlapping coronal acquisitions to cover most of the cerebral hemispheres.
  - Whole-brain T2-FLAIR scan (coronal 3D SPACE sequence, field-of-view 235 mm^2^, matrix 512 × 512, reconstructed in-plane resolution 0.46 mm^2^, 176 1 mm sections, TR/TE/TI = 4,800/354/1,800 msec, nonselective inversion pulse, echo-train length 298, bandwidth 780 Hz/pixel, acceleration factor 2, acquisition time 14 min).
  - Whole-brain T1-SPACE (axial 3D acquisition, acquisition matrix 256 × 256, isotropic resolution 0.9 mm, 112 sections, TR/TE = 600/20 msec, flip angle 120°, echo-train length 28, acquisition time 10 min).
4. Inject MRI contrast agent, either gadobutrol (0.1 mmol/kg body weight, i.v., Bayer HealthCare) or another standard agent, at a rate of 0.3 ml/min followed by 10 ml of saline flush.
5. Repeat MRI sequences A3a, A3c, and A3d after completion of the infusion.
6. Covert scanner-generated DICOM images into NIFTI files for processing using dcm2nii script (nitrc.org, open source).
7. Co-register pre- and post-contrast images, perform skull-stripping, and subtract pre-contrast images from post-contrast images using standard algorithms implemented in MIPAV software (select Algorithms/Registration/Optimized Automatic Registration and Utilities/Image Calculator/Subtract, respectively).
8. Import subtraction images into OsiriX software for maximum intensity projection (MIP) 3D rendering (select 2D/3D and then 3D Surface Rendering). (Figure 2)
9. For more specific lymphatic imaging, perform Steps B1-B8 using gadofosveset (0.03 mmol/kg body weight, i.v., Lantheus Medical Imaging) rather than gadobutrol. Compare the subtraction images obtained from gadofovest and gadobutrol experiments to identify the lymphatic vessels (Figure 3).

### C. Immunohistochemistry, single staining

**Figure 4:**
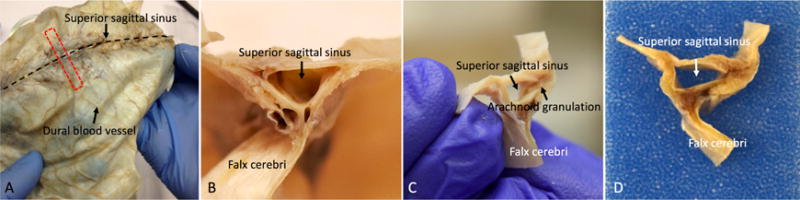
Whole-mount and coronal sections of the human dura mater for histological analysis A. The red dotted line shows the sampling direction. B, C, and D. Show the coronal view of the dura mater sample before tissue processing.

1. Fix freshly dissected human dura mater with 10% formalin for 24-48 h at room temperature. Commercial 10% neutral buffered formalin (NBF) contains a small percentage of methanol as a stabilizer, which is not a problem for the majority of procedures. Dura should be fixed as soon as possible using gentle agitation (swirling) of the specimen to aid penetration and fixation reaction. Tissue should be fixed for 24-48 h in NBF, and then stored in 1× PBS with a few drops of 10% formalin at room temperature.
2. Trim the dura into coronal sections and embed the tissue in a paraffin block (see Figure 4). Our recommendation is to focus on the coronal sections near the superior sagittal sinus, which can be easily identified in the dura.
3. Using rotary microtome, cut the paraffin-embedded tissue block into sections of 3-8 μm thickness. Float the sections in 20% ethanol at room temperature, then transfer them to a 44 °C water bath. (see Note 1 and Video 1)
4. Transfer the sections onto Superfrost Plus Microslides, as uncoated or uncharged slides may not retain the tissue. Before drying out the slides, remove residual water using a snap of the wrist (imagine wielding a whip), which is important to prevent sections from lifting from slides. Allow the slides to dry vertically overnight, at room temperature, to allow trapped water to escape downward.
5. Deparaffinize slides using xylene (3 changes of xylene, each 3 min).
6. Rehydrate slides using 100% alcohol (3 changes, each 3 min), 80% alcohol (3 min) and 50% alcohols (3 min), respectively.
7. Rinse slides in deionized water for 1 min.
8. Perform heat-induced antigen retrieval to unmask the antigenic epitope using a steamer. Add tap water to the water base, to the “Max” line, and put the steaming plate onto the water reservoir. Fill a plastic Coplin jar with Target Retrieval Solution or Target Retrieval Solution, at pH 9, and dip deparaffinized/rehydrated slides in the jar. Place the plastic Coplin jar in the steamer and cover it. Turn on the steamer and set the timer for 20 min to incubate it at 95-100 °C. We recommend steamer for heat-induced antigen retrieval instead of microwave or pressure cooker, because it reduces the chance of the section falling off the slide.
9. Take out the Coplin jar and allow it to cool down for 10 min at room temperature.
10. Rinse slides gently in Tris-buffed saline (TBS) for 5 min. Use TBS or TBS-0.5% Tween 20 (TBST) during slide washing to prevent sections from falling off.
11. Immerse sections in 0.3% H2O2 solution in deionized water at room temperature for 10 min to block endogenous peroxidase activity.
12. Rinse slides gently in TBST for 1 min.
13. Draw the hydrophobic barrier around the tissues using PAP pen.
14. Rinse slides gently in TBST 20 for 1 min.
15. Drop 3-4 of Dako Protein Block on the tissue and incubate at room temperature for 20 min in a humidified chamber.
16. Gently drop off the excess Dako Protein Block from the slides. Do not rinse the slides in this step.
17. Apply primary antibody + Dako Antibody Diluent (see Table 1 for antibody dilution factor; 100- 200 μl is required to cover the tissue) on the tissues, and incubate at room temperature for 2 h or at 4 °C overnight in a humidified chamber. Make sure that the antibody is spread well on the tissues.
18. Wash slides in TBST 3 times, 5 min each, using a rocker.
19. Apply secondary antibody (HRP anti-Mouse IgG or HRP anti-rabbit IgG) on the tissues and incubate for 30 min at room temperature in a humidified chamber.
20. Wash slides in TBST 3 times, 5 min each, using a rocker.
21. Drip 3-4 drops of freshly made DAB substrate solution on the slide and check the brown color of antibody signal by microscopy.
22. If the staining reveals adequate intensity, stop the DAB reaction by dipping slides in deionized water. Over-staining will lead to high background that will obscure the true signals.
23. Dip slides in Leica Hematoxylin 560 MX for 10 sec, for better morphology and contrast.
24. Rinse slides in tap water for 5 min.
25. Immerse slides in bluing solution (Leica Blue buffer or 0.2% ammonia solution or 0.1% lithium carbonate solution).
26. Dehydrate slides through air dry and coverslip using Permount mounting solution. The mounted slides can be kept at room temperature constantly.

### D. Immunohistochemistry, double staining of D2-40 and CD31 (simultaneous double staining of lymphatic and blood vessels, respectively)

1. Follow Steps C5-C16 above.
2. Apply cocktails of primary antibodies + Dako Antibody Diluent on the tissues and incubate at room temperature for 2 h in a humidified chamber.
3. Wash slides in TBST 3 times, 5 min each, using a rocker.
4. Apply secondary antibody (HRP anti-Mouse IgG for D2-40 and ImmPRESS™-AP anti-Rabbit IgG for CD31) on the tissues and incubate for 30 min at room temperature in a humidified chamber.
5. Wash slides in TBST 3 times, 5 min each, using a rocker.
6. Drip 3-4 drops of freshly made DAB substrate solution on the slide and check the brown color of D2-40 antibody signal by microscopy.
7. Wash slides in deionized water to stop the DAB reaction.
8. Drip 3-4 drops of fresh Vector Blue substrate solution on the same slide and check the blue color of CD-31 antibody signal by microscopy.
9. If the staining reveals enough intensity, stop the Vector Blue reaction by dipping slides in deionized water.
  CAUTION: Do NOT perform hematoxylin counterstaining following use of the Vector Blue chromogen.
10. Dehydrate slides through air dry and coverslip using Permount mounting solution.

### E. Immunohistochemistry, double staining of PROX1 and CD31 (sequential double staining)

1. Follow Steps C5-C22 above. Finish PROX1 immunostaining without counterstaining.
2. Drip 3-4 drops of Dako Protein Block on the tissue and incubate at room temperature for 20 min in a humidified chamber.
3. Gently drop off the excess Dako Protein Block from the slides. Do not rinse the slides in this step.
4. Apply CD31 antibodies + Dako Antibody Diluent on the tissues and incubate at room temperature for 2 h in a humidified chamber.
5. Wash slides in TBST 3 times, 5 min each, using a rocker.
6. Apply secondary antibody (ImmPRESS™-AP anti-Rabbit IgG for CD31) on the tissues and incubate for 30 min at room temperature in a humidified chamber.
7. Wash slides in TBST 3 times, 5 min each, using a rocker.
8. Drip 3-4 drops of fresh Vector Blue substrate solution on the same slides and check the blue color of CD-31 antibody signal by microscopy.
9. Stop the Vector Blue reaction by dipping slides in deionized water if the staining reveals enough intensity.
  CAUTION: Do NOT perform hematoxylin counterstaining following use of the Vector Blue chromogen.
10. Dehydrate slides through air drying and coverslip using Permount mounting solution.

### F. Immunofluorescence, double staining of D2-40 + CD31 (simultaneous double staining)

1. Follow Steps D1-D3 above.
2. Apply cocktails of secondary antibodies (Goat anti-Mouse IgG Alexa Fluor 488 and Goat anti- rabbit IgG Fluor 594, 1:200 diluted in Dako Antibody Diluent) on the tissues and incubate for 30 min at room temperature in a humidified chamber.
3. Wash slides in TBST 3 times, 5 min each, using a rocker.
4. Dehydrate slides through air dry and coverslip using Fluoro-Gel II Mounting Medium.
5. Observe the localization of D2-40 and CD31 with fluorescence microscopy.

## Data analysis

**Figure 5:**
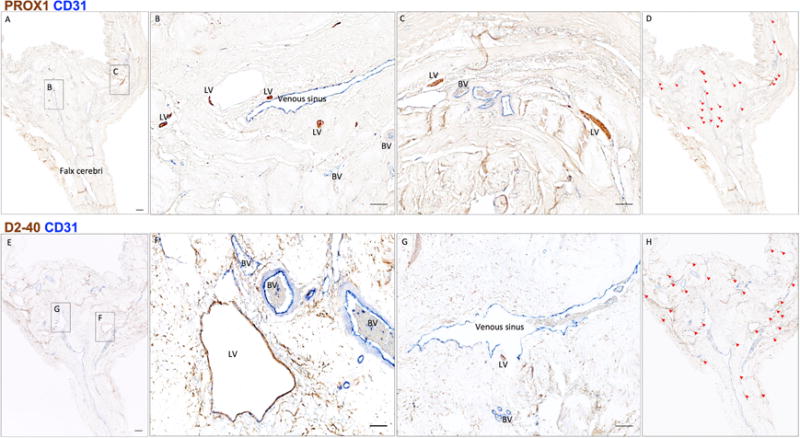
Neuropathology of human dural lymphatic vessels, coronal section A, B and C. Within the dura mater, lymphatic and blood vessels can be differentiated using double staining for PROX1 (a transcription factor involved in lymphangiogenesis, nuclear staining) and CD31 (a vascular endothelial cell marker). E, F and G. Similarly, lymphatic and blood vessels can be differentiated using double staining for D2-40 (endothelial membrane staining) and CD31. Red blood cells are seen within blood vessels, but not within lymphatic vessels. D and H. Using Zeiss Zen Blue software, lymphatic structures are marked on the digitalized slide. Insets (B, C, F, G) were rotated relative to the original Figures in A and E. Scale bars: 1 mm (A, G), 100 μm (B, C, F, G). Abbreviations: LV–lymphatic vessels; BV–blood vessels. (Modified from Figure 3 in Absinta *et al*. [2017]. Creative Commons Attribution License)

Scan the entire slide and stitch it together by greater than 10× magnification using Zeiss Microscope, camera, and Zeiss Zen Blue software. On slides double-stained for lymphatic and vascular endothelial markers (D2-40/CD31 and PROX1/CD31), identify lymphatic structures and mark them on the screen under the microscope using the following criteria: (a) structures of endothelial cell- lined vessel; (b) vessel with thin endothelial cells, the nuclei of cell bulge into the lumen; (c) semi- collapsed thin vessel wall with poor basal lamina; and (d) no or only a few red blood cells in the lumen of the vessel (Killer *et al*., 2008). Lymphatic vessels are counted, and their dimensions are measured. If samples vary in disease type or treatment status, simple comparative statistics may be computed on the count and diameter data (Figure 5).

## Notes

1. Human dura mater is a very tough tissue, and microtome sectioning is difficult. Chilling the paraffin blocks (*e.g.*, Leica RM Cool Clamp™) makes sectioning of dura easier. Also, when tissue is exposed on the surface of a paraffin block by rough trimming, it has the capacity to absorb water, which can penetrate a small distance into the tissue, resulting in softening and swelling it. For the dura mater, this effect may allow a couple of sections to be cut easily. By placing the trimmed block surface on melting ice or in a tray of ice water at 4 °C for 1 min, followed by use of a cold wet paper towel for 30 sec to 1 min, the sectioning becomes easier. Generally, after this procedure, the best quality sections are achieved by cutting very slowly.
2. Paraffin sections of dura may wrinkle easily, which can generate artifacts and ultimately nonspecific staining. Non-standard flotation techniques may be useful if the sections obtained from a block are highly wrinkled. If sections are initially floated in 20% ethanol then transferred, on a slide, to a hot flotation bath, the wrinkling may be mitigated. 20% ethanol actively removes the wrinkles out because it has lower surface tension than water.
3. Formalin fixed-paraffin embedded (FFPE) human skin can be used as a positive control for lymphatic vessel marker and assessment. FFPE Hippocampus (CA3) of brain tissue can be used as good positive control for PROX1 staining.

## Recipes

1. TBS-0.5% Tween 20 (TBST)
  200 ml 10× TBS
  1,800 ml deionized water
  Add 1 ml of Tween 20, mixed well using a magnetic stirrer

## Figures and Tables

**Video 1:**
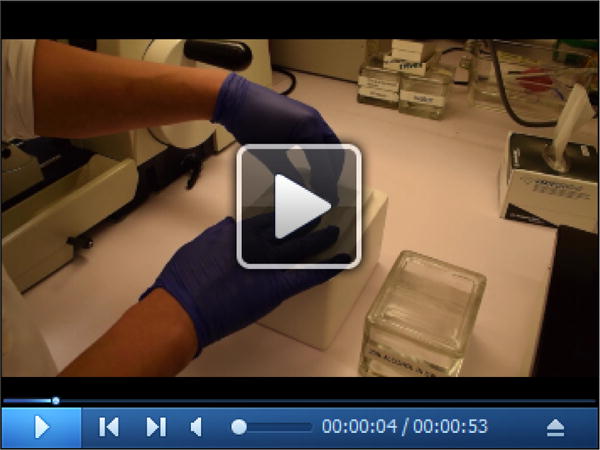
Demonstration of the sectioning of the human dura mater using a microtome Before sectioning, place the paraffin tissue block surface on melting ice or cold wet paper towel. After sectioning, place the section in 20% ethanol and then into a warm floating bath.

**Table 1:** Condition of antigen retrieval, antibody dilution and time of incubation

1^st^ Antibody	Function	Antigen retrieval	Antibody dilution and time of incubation
LYVE1	Lymphatic endothelialcells	Target Retrieval Solution, pH 9 20 min by steamer	1:200, 4 °C overnight
Podoplanin (D2-40)	Lymphatic endothelialcells	Target Retrieval Solution, pH 9 20 min by steamer	1:50, 2 h RT
CD31	Blood endothelial cells	Target Retrieval Solution, pH 9 20 min by steamer	1:50, 2 h RT
PROX1	Lymphatic endothelial nuclear transcription factor	Target Retrieval Solution, 20 min by steamer	1:300, 4 °C overnight
COUP-TF II	Lymphatic endothelial nuclear transcription factor	Target Retrieval Solution, 20 min by steamer	1:200, 4 °C overnight
CCL21	Lymphatic endothelial cells	Target Retrieval Solution, pH 9 20 min by steamer	1:200, 1 h RT

## References

[R1] Absinta M, Ha SK, Nair G, Sati P, Luciano NJ, Palisoc M, Louveau A, Zaghloul KA, Pittaluga S, Kipnis J, Reich DS (2017). Human and nonhuman primate meninges harbor lymphatic vessels that
can be visualized noninvasively by MRI. eLife.

[R2] Aspelund A, Antila S, Proulx ST, Karlsen TV, Karaman S, Detmar M, Wiig H, Alitalo K (2015). A dural lymphatic vascular system that drains brain interstitial
fluid and macromolecules. J Exp Med.

[R3] Louveau A, Smirnov I, Keyes TJ, Eccles JD, Rouhani SJ, Peske JD, Derecki NC, Castle D, Mandell JW, Lee KS, Harris TH, Kipnis J (2015). Structural and functional features of central nervous system
lymphatic vessels. Nature.

[R4] Killer HE, Jaggi GP, Miller NR, Flammer J, Meyer P (2008). Does immunohistochemistry allow easy detection of lymphatics in
the optic nerve sheath?. J Histochem Cytochem.

